# Ischemia-induced Neuronal Cell Death Is Mediated by Chemokine Receptor CX3CR1

**DOI:** 10.1038/s41598-017-18774-0

**Published:** 2018-01-11

**Authors:** Jinkun Wang, Yan Gan, Pengcheng Han, Junxiang Yin, Qingwei Liu, Soha Ghanian, Feng Gao, Guanghui Gong, Zhiwei Tang

**Affiliations:** 1 Department of Neurosurgery, The First Affiliated Hospital of Kunming Medical University, Kunming, Yunnan China; 2 Department of Neurology, Barrow Neurological Institute, St. Joseph’s Hospital and Medical Center, Phoenix, AZ USA; 3 Department of Radiology, Barrow Neurological Institute, St. Joseph’s Hospital and Medical Center, Phoenix, AZ USA; 4 Brown University Warren Alpert Medical School, Providence, RI USA

## Abstract

The chemokine fractalkine (CX3CL1) and its receptor CX3CR1 play a fundamental role in the pathophysiology of stroke. Previous studies have focused on a paracrine interaction between neurons that produce fractalkine and microglia that express CX3CR1 receptors in the central nervous system. Recent findings have demonstrated the functional expression of CX3CR1 receptors by hippocampal neurons, suggesting their involvement in neuroprotective and neurodegenerative actions. To elucidate the roles of neuronal CX3CR1 in neurodegeneration induced by ischemic stroke, a mouse model of permanent middle cerebral artery occlusion (pMCAO) was employed. In the pMCAO mice, increased CX3CR1 levels, apoptosis-associated morphological changes, and Caspase 3-positive neuronal cells were observed in the striatum and in the hippocampus 24 hours after occlusion. Upregulation of CX3CR1 in ischemic neurons is associated with neuronal apoptotic cell death. In contrast, ischemia-induced apoptotic neuronal cell death was decreased in CX3CR1 deficient mice. Cultured primary hippocampal neurons obtained from CX3CR1 deficient mice were more resistant to glutamate-induced excitotoxicity by blocking calcium influx than those from wild-type mice. For the first time, we reported that neuronal CXCR1 mediates neuronal apoptotic cell death in ischemia. Our results suggest that modulating CXCR1 activity offers a novel therapeutic strategy for stroke.

## Introduction

Ischemic stroke is the fourth leading cause of death in the United States and Europe, and it is a major cause of adult disability^1^. During an ischemic event, impaired blood flow does not allow the delivery of oxygen and glucose, leading to energy depletion, over-activation of glutamate receptors and release of excess glutamate, increase of intracellular calcium, loss of membrane potential and cell depolarization, and, eventually, cell death^2,3^. The need for better stroke therapies has encouraged research in the cellular and molecular mechanisms of ischemic brain damage, with a major focus on the neuronal response under ischemic conditions.

The chemokine fractalkine (CX3CL1)/CX3C chemokine receptor 1 (CX3CR1) system has been shown to play an important role in modulating the communication between neurons and resident microglia in the central nervous system (CNS)^4–6^. It has been previously reported that CX3CR1 deficiency is associated with decreased neuronal apoptosis and improved outcomes following ischemic brain injury^7,8^. The underlying mechanism has focused on the signaling from neurons to microglia specific to microglia activation, survival, proliferation, and neurotoxicity, since the expression of fractalkine in the brain has been found to be widespread and localized mainly to neurons^9^, whereas the expression of its G protein-coupled receptor CX3CR1 is thought to be restricted to microglia and astrocytes^10^. Meucci *et al*. recently provided direct evidence for the functional expression of CX3CR1 receptors by hippocampal neurons and demonstrated their involvement in the neuroprotective action of fractalkine^11^. CX3CR1 immunoreactivity was also transiently detected in neurons, particularly in CA1 pyramidal cells, in a rat model of pilocarpine-induced status epilepticus^12^.

We hypothesized that CX3CR1 directly mediates brain ischemia-induced neuronal apoptosis. We now report that CX3CR1 is upregulated in ischemic neurons in mice subjected to focal cerebral ischemia. Higher levels of CX3CR1 are associated with apoptotic neuronal death, and calcium influx plays a crucial role in this process of cell death.

## Results

### Cultured hippocampal neurons express CX3CR1

**Figure 1:**
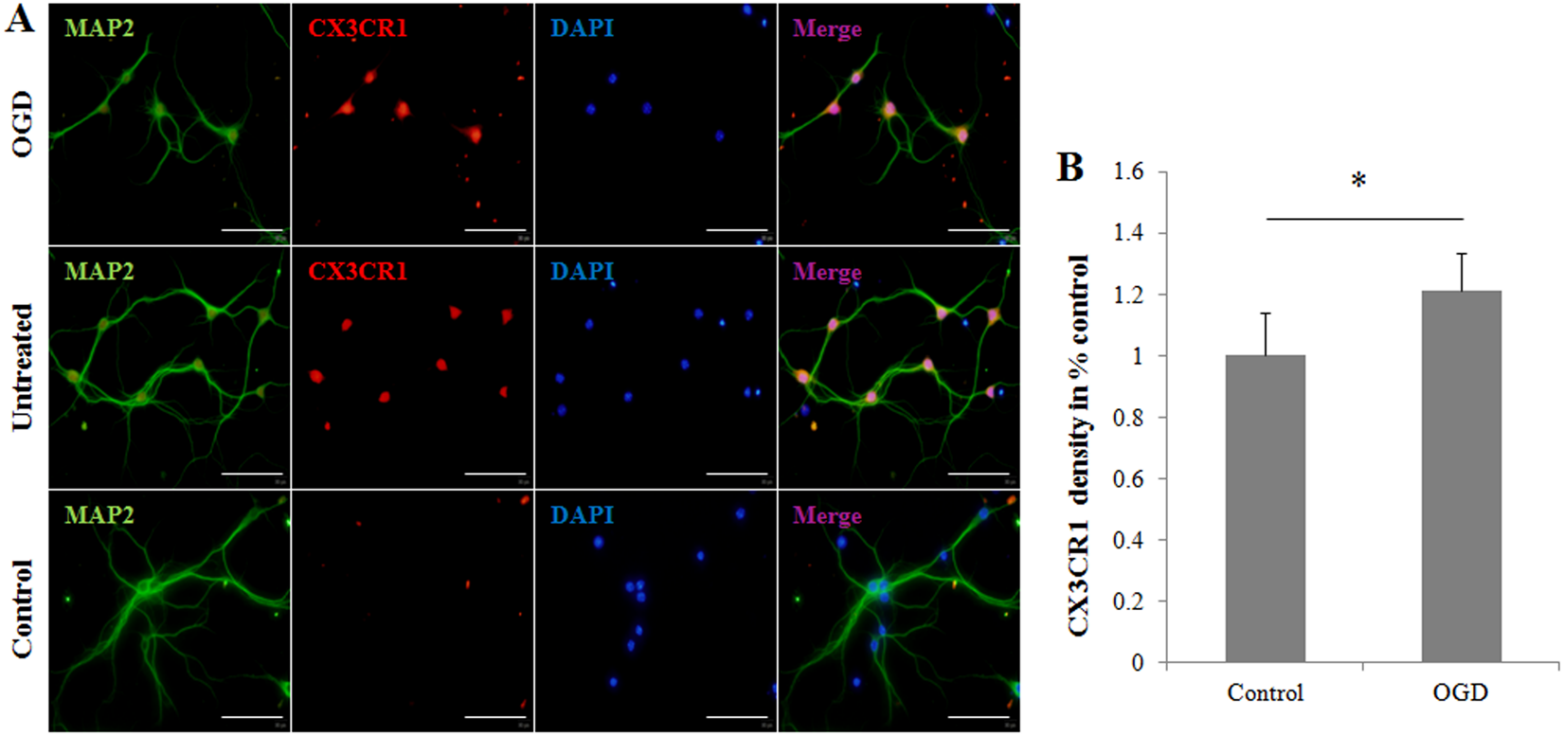
Postnatal microglia-depleted neuronal cultures express CX3CR1 and overexpress CX3CR1 under OGD. (**A)** Mature hippocampal cultures prepared from postnatal C57BL/6 mice and treated with 2.5 μM cytosine arabinoside (AraC) were stained with anti-CX3CR1 (red) and anti-MAP2 (green). CX3CR1 significantly co-localizes with MAP2^+^ cell bodies in normal hippocampal culture (middle panel). Compared to normal culture conditions, OGD-treated neurons overexpressed CX3CR1 (upper panel). Normal cultures without CX3CR1 staining served as experimental controls (lower panel). Scale bars = 100 µm, n = 4/group. (**B**) CX3CR1 expression was evaluated by calculating the mean density with the VisionWorks^®^LS Image Acquisition and Analysis Software (UVP, LLC, CA, USA).

Neuronal CX3CR1 expression in mice is controversial^13–16^ and has not yet been investigated in our system; we first assayed mature microglia-depleted hippocampal cultures from postnatal C56BL/6 mice for CX3CR1 expression. Microglial depletion was attained using a rigorous densitometric segregating centrifugation protocol, and it was ensured for the lifespan of the culture by treatment with 2.5 μM cytosine arabinoside (AraC) on days *in vitro* (DIV) 3. We found that CX3CR1 expression significantly co-localized with microtubule-associated protein (MAP) 2-positive cell bodies in hippocampal cultures (Fig. 1, middle panel). Using oxygen-glucose deprivation (OGD) as an *in vitro* ischemia model, we found that cultured hippocampal neurons under ischemia expressed higher CXCR1 than untreated ones (Fig. 1, upper panel). These results indicate that CX3CR1-positive neurons within our system of mature, microglia-deficient neuronal cultures expressed CX3CR1 protein and that the degree of protein expression was unregulated under ischemic conditions.

### Upregulation of CX3CR1 on ischemic neurons in pMCAO mice

**Figure 2:**
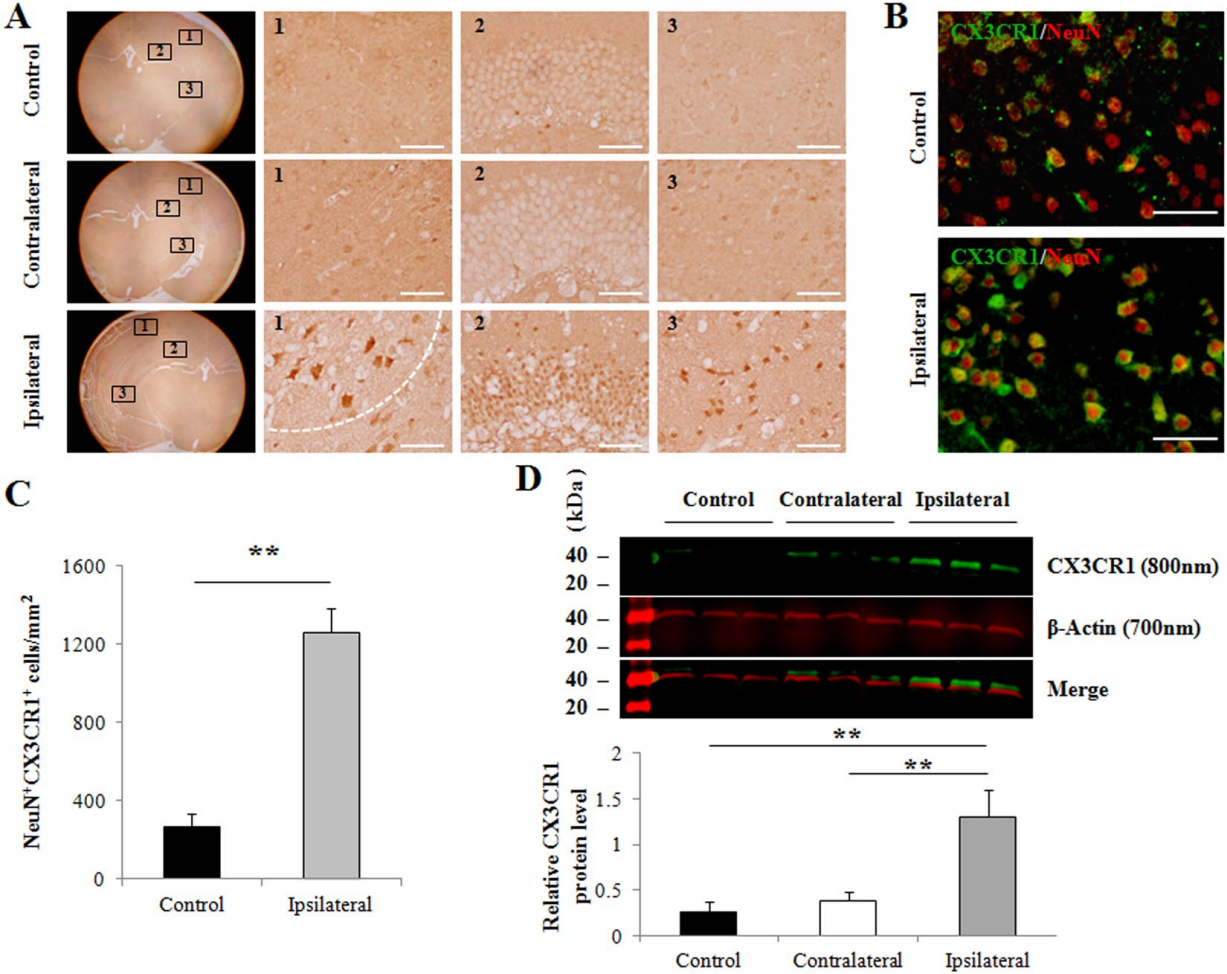
CX3CR1 was upregulated in neurons of the infarct brain in pMCAO mice. (**A**) Representative CX3CR1 stains in the peri-infarction (1), hippocampus (2), and striatum (3) of the ipsilateral hemisphere (bottom panel), the counterparts in the contralateral hemisphere (middle panel), and the intact control hemisphere (upper panel) in C57BL/6 mice 24 hours after pMCAO. (**B**) Double staining with CX3CR1 and NeuN demonstrated that CX3CR1 was mainly expressed in ischemic neurons in the infarct. (**C**) Semi-quantification analysis showed that the number of CX3CR1/NeuN positive cells significantly increased 24 hours after pMCAO (**p < 0.01). Cells were counted in 5 randomly chosen 200× magnification fields on five sections in four replicate mice per group in three separate experiments. (**D**) CX3CR1 protein expression in brain ischemia. Western blot analysis was performed using brain homogenate obtained from the ipsilateral, contralateral, and intact control hemispheres 24 hours after pMCAO. The results were quantified and normalized to β-Actin. Scale bars = 100 µm, n = 4/group.

To assess CX3CR1 expression in ischemic neurons, histological analysis with CX3CR1 staining was performed in brain slices from pMCAO C57BL/6 mice, a global cerebral ischemia model with striatal and cortical damage. No apparent changes in CX3CR1 levels were seen in the contralateral hemispheres compared to intact controls (Fig. 2A, upper and middle panel). CX3CR1 expression was elevated in the peri-infarct, hippocampus, and striatum of the ipsilateral hemisphere 24 hours after occlusion (Fig. 2A, bottom panel). Double staining with NeuN and CX3CR1 was performed and confirmed that CX3CR1 was upregulated in ipsilateral ischemia compared to the intact control, and co-localized with NeuN in most cells (Fig. 2B,C). An upregulation of CX3CR1 was also detected in microglia but at a relatively lower level within 24 hours and showed a peak at 72 hours after occlusion (Supplementary Fig. S1). Elevated expression of CX3CR1 protein in the infarct was confirmed by Western blot (Fig. 2D).

### Coupling of CX3CR1 to apoptotic neuronal cell death

**Figure 3:**
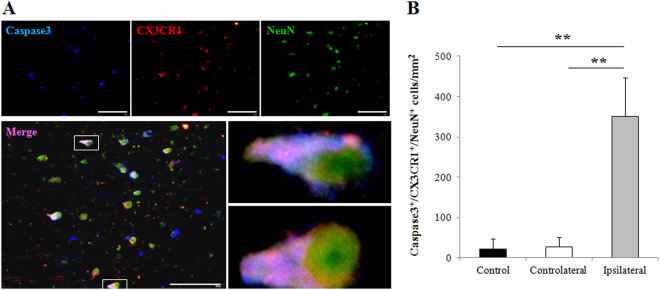
Coupling of CX3CR1 to apoptotic neuronal cell death in pMCAO mice. (**A**) Triple staining with cleaved Caspase-3 (apoptotic marker), CX3CR1, and NeuN (neuron marker) was performed and demonstrated that CX3CR1 was coupled to apoptotic neurons in ischemic damage 24 hours following pMCAO. Scale bars = 100 µm, n = 4/group. (**B**) Semi-quantification analysis showed that the number of Caspase-3/CX3CR1/NeuN positive cells was significantly increased in the ipsilateral hemisphere (**p < 0.01 vs. contralateral and intact control), whereas no difference was observed in these sites between the contralateral and control hemispheres. Cells were counted in 5 randomly chosen 200× magnification fields on five sections in four replicate mice per group in three separate experiments.

Next, we investigated whether CX3CR1 upregulation in the infarct 24 hours following pMCAO was associated with ischemia-induced neuronal apoptosis by assessing co-expression of cleaved Caspase-3 (apoptotic marker), CX3CR1, and NeuN (neuron marker) in the infarct. Triple immunofluorescence staining demonstrated that CX3CR1 is co-localized in most apoptotic neurons in ischemia (Fig. 3A). Compared to the contralateral and intact controls, the infarct areas showed an increase in Caspase-3/CX3CR1/NeuN positive cells (Fig. 3B).

### Disruption of the CX3CR1 gene protects ischemia-induced neuronal cell death

**Figure 4:**
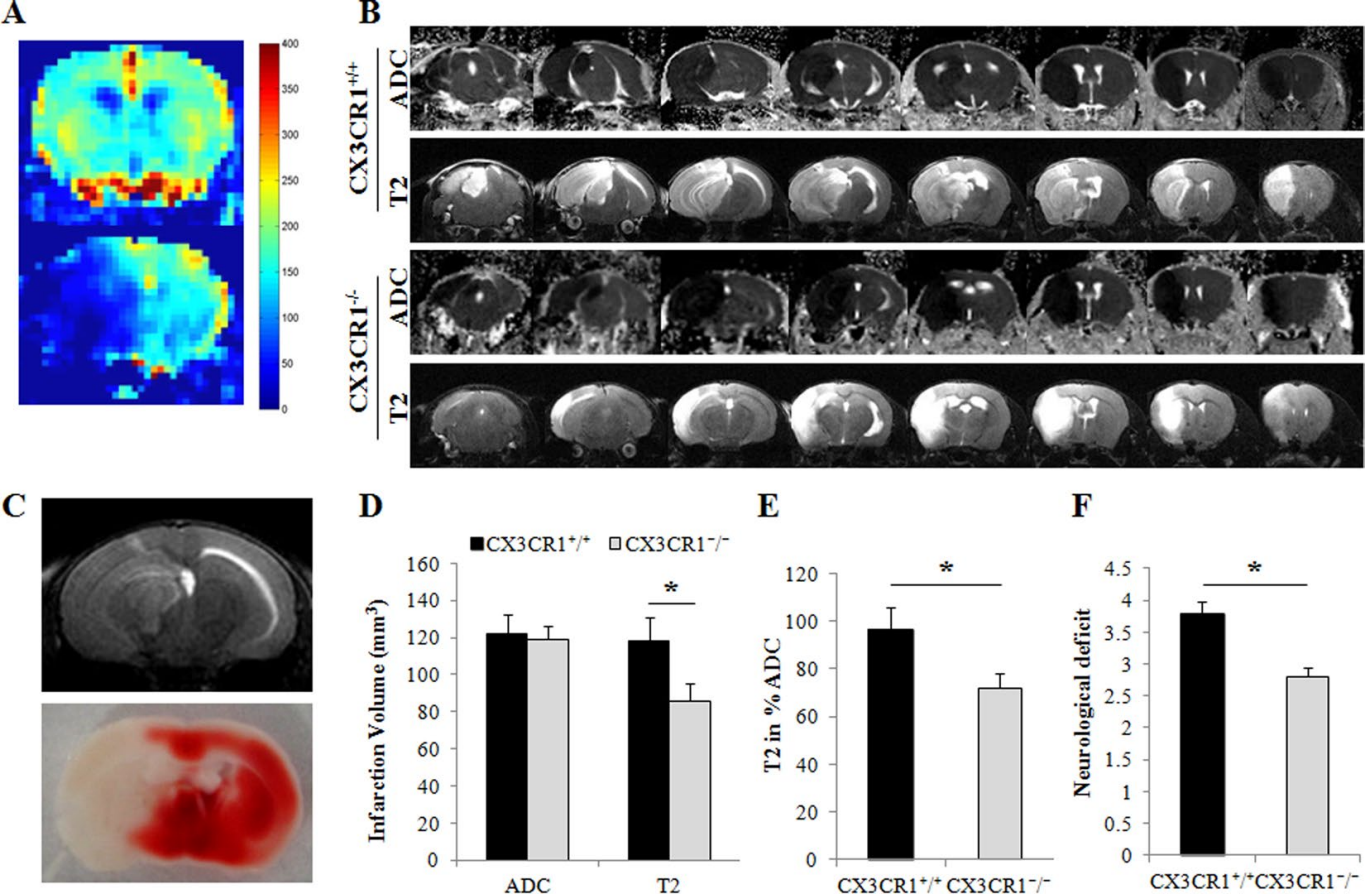
CX3CR1 deficiency attenuated infarct volume and neurological deficit after pMCAO. (**A**) Cerebral blood flow (CBF) analysis showed that the blood flow of the right middle cerebral artery territory decreased dramatically after occlusion (normal 168.3 ± 8.5 ml/100 g/min vs. pMCAO 37.3 ± 10.3 ml/100 g/min). There was no difference between the CX3CR1^+/+^ and CX3CR1^−/−^ groups. (**B**) Apparent diffusion coefficient (ADC) and T2-weighted MRI imaging, respectively, showed the damaged volume at 30 min and the infarction volume 24 hours after pMCAO between the CX3CR1^+/+^ and CX3CR1^−/−^ groups. (**C**) TTC staining demonstrated the same infarction volume as seen in the T2 images. (**D**) Quantification analysis showed the damaged volume was similar between the two groups 30 min post-pMCAO, whereas the infarction volume 24 hours post-pMCAO was significantly smaller in CX3CR1^−/−^ mice (*p < 0.05 vs. CX3CR1^+/+^). (**E**) Among damaged brains with similar ADC, 96.7% of the CX3CR1^+/+^ group versus 71.9% of the CX3CR1^−/−^ group proceeded to ischemia in 24 hours, as shown in the T2-weighted MRI. (**F**) CX3CR1 deficiency relieved the neurological deficits in mice 24 hours after ischemia (*p < 0.05). Representative images were shown; n = 9–13 per group.

To examine the role of neuronal CX3CR1 in ischemia-induced neuronal cell death *in vivo*, we used CX3CR1^−/−^ mice. Figure 4 shows the physiological parameters during pMCAO in the WT (CX3CR1^+/+^) and CX3CR1^−/−^ mice. There was no significant difference between the WT and CX3CR1^−/−^ mice in the decrease of regional cerebral blood flow (CBF) (Fig. 4A). The apparent diffusion coefficient (ADC), measuring the magnitude of diffusion of water molecules within cerebral tissue, was acquired 30 min after pMCAO to assess the volume of damage in the ipsilateral hemisphere. No significant differences in damage volume were observed between CX3CR1^−/−^ and WT mice (Fig. 4B and D). The infarct volume, assessed by MRI T2-weighted images 24 hours after pMCAO, was markedly smaller in CX3CR1^−/−^ mice relative to WT mice (Fig. 4B and D, p < 0.05). The infarct observed in the MRI scan was confirmed by 2,3,5-triphenyltetrazolium chloride (TTC) staining (Fig. 4C). Given the same early damage indicated by the magnitude of the diffusion of water molecules within the tissues shown in ADC, approximately 96.7% of the WT brains proceeded to ischemia damage as shown in T2-weighted MRI; this occurrence was only seen in 71.9% of CX3CR1^−/−^ brains (Fig. 4E, p < 0.05). To further determine the differential response to pMCAO between CX3CR1^−/−^ and WT mice, the neurological deficit was assessed. The average clinical score was 3.8 ± 0.4 in WT mice and 2.8 ± 0.3 in CX3CR1^−/−^ mice 24 hours following pMCAO (Fig. 4F, p < 0.05).

**Figure 5:**
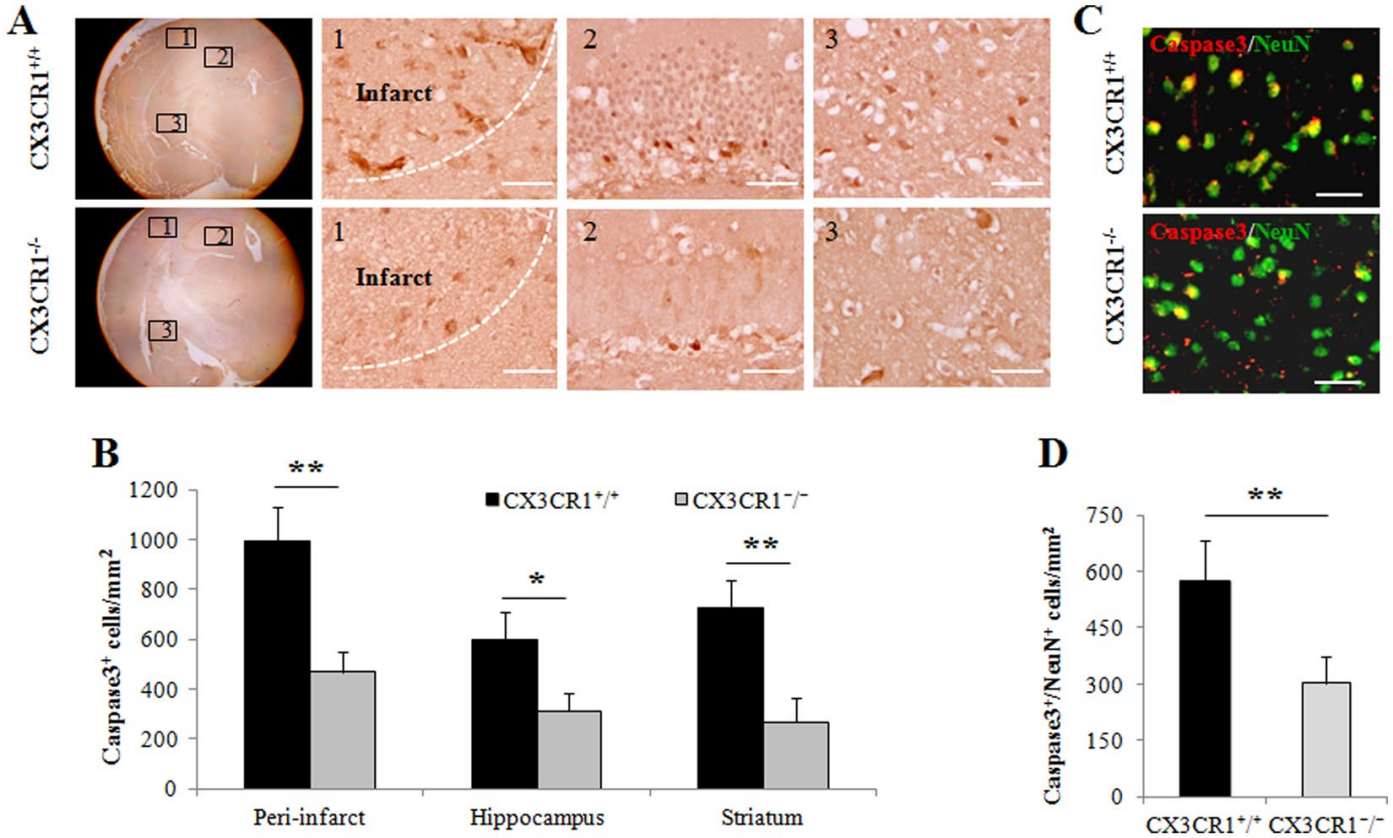
CX3CR1 deficiency decreased Caspase-3 positive neuronal cells after pMCAO. (**A**) Immunohistochemical staining of Caspase-3 antibody in the ipsilateral (1) peri-infarct region, (2) hippocampus, and (3) striatum 24 hours after pMCAO. (**B**) Semi-quantification analysis of the number of Caspase-3 positive cells demonstrated that CX3CR1 deficiency decreased the number of apoptotic cells in the peri-infarction region, hippocampus, and striatum (*p < 0.05, **p < 0.01 vs. CX3CR1^+/+^ group). (**C**) Double staining with Caspase-3 and NeuN showed that CX3CR1^−/−^ mice had fewer apoptotic neurons in the infarct than CX3CR1^+/+^ mice after pMCAO. (**D**) Semi-quantification analysis of Caspase-3/NeuN-positive cells demonstrated that CX3CR1 deficiency decreased the number of the apoptotic neurons in the infarct region (**p < 0.01 vs. CX3CR1^+/+^ group). Representative images were shown; scale bars = 50 µm, n = 4/group. Cells were counted in 5 randomly chosen 200× magnification fields on five sections from mice in three independent experiments.

We analyzed cell death in the peri-infarct, hippocampus, and striatum of WT and CX3CR1^−/−^ pMCAO mice by immunostaining the cleaved Caspase-3. Cells positive for Caspase-3 were observed in these three locations of the ischemic brain of CX3CR1^+/+^ mice (Fig. 5A, upper panel). In contrast, this increase in apoptotic cells was markedly attenuated in CX3CR1^−/−^ mice (Fig. 5A, lower panel). The number of Caspase-3-positive cells in each area of the ischemic hemisphere was counted, and the average number was significantly lower in CX3CR1^−/−^ mice after pMCAO (Fig. 5B). Further, we found that Caspase-3 significantly co-localized with NeuN-positive cells in the ipsilateral brain (Fig. 5C). Cell counts of NeuN- and Caspase-3-positive cells indicated that CX3CR1 deficiency decreased the number of apoptotic neurons in the infarct region (Fig. 5D).

### Primary neurons from CX3CR1^−/−^ mice are resistant to glutamate-induced cell death by blocking calcium influx

**Figure 6:**
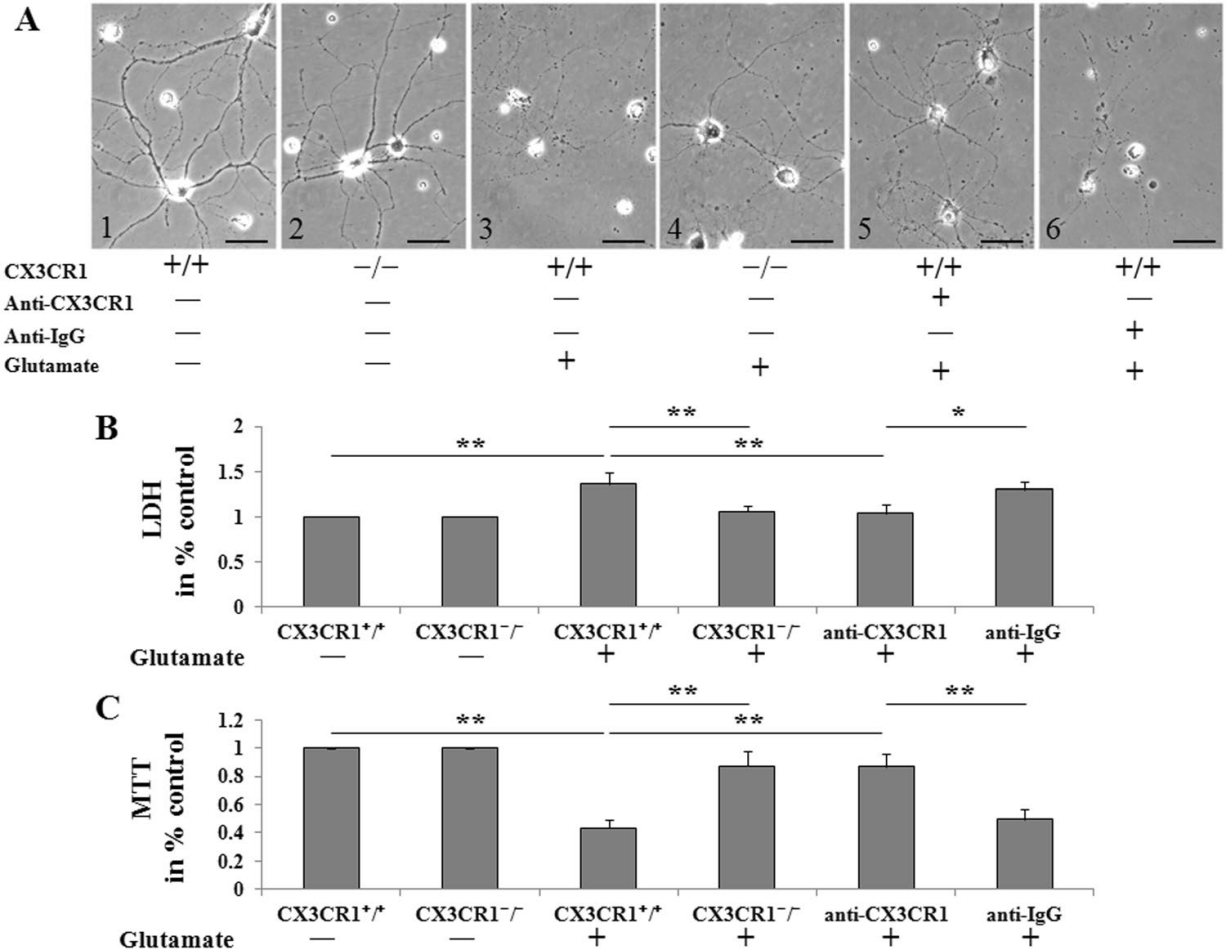
CX3CR1 deficiency protected neurons against glutamate-induced apoptosis *in vitro*. (**A**) Morphological changes of neurons after exposure to glutamate for 30 min. (1 and 2) Cultured healthy CX3CR1^+/+^ and CX3CR1^−/−^ neurons extended several dendritic trunks, and the dendrites had numerous protrusions. (3) Glutamate treatment caused the rupture of the dendrites and shrinking of the cell body in CX3CR1^+/+^ neurons, whereas (4) CX3CR1 deficiency protected the neurons against this damage, resulting in the induced formation of bead-like structures and a decrease of fine protrusions only. (5) Neutralizing the CX3CR1 with anti-CX3CR1 antibody could attenuate the damage of glutamate, (6) while the same animal source of anti-IgG antibody could not attenuate the damage. (**B**) Cell death was assessed by measuring LDH release from neurons in all groups. Glutamate treatment could increase the LDH release, whereas CX3CR1 deficiency can attenuate the increase of LDH. Moreover, neutralizing the CX3CR1 could also attenuate this increase of LDH. (**C**) An MTT assay was performed to evaluate neuronal survival after glutamate treatment. CX3CR1 deficiency and neutralization could reduce glutamate-induced neuronal death (*p < 0.05, **p < 0.01). Representative images were shown; scale bars = 50 µm, n = 10/group.

Neuronal injury caused by ischemia after occlusion of cerebral arteries is believed to be mediated by excessive activation of glutamate receptors. In the ischemic brain, levels of extracellular glutamate increase shortly after the onset of ischemia. Overactivation of glutamate receptors in postsynaptic spines induces neuronal apoptosis. We examined the effects of glutamate on primary cultured neurons from WT and CX3CR1^−/−^ mice. Cultured healthy control neurons extended several dendritic trunks, and the dendrites had numerous protrusions (Fig. 6A-1,2). After exposure to glutamate (50 μM glutamate + 10 μM glycine) for 30 min, rupture of the dendrites and a reduction in the size of the cell body were observed in CX3CR1^+/+^ neurons (Fig. 6A-3). CX3CR1 deficiency protected neurons against this damage, resulting in the induced formation of bead-like structures and a decrease in fine protrusions but not rupture of the cell body or dendrites (Fig. 6A-4). Neutralizing the CX3CR1 with anti-CX3CR1 antibody can also attenuate glutamate-induced damage in CX3CR1^+/+^ neurons (Fig. 6A-5,6). The LDH and MTT assays were further employed to investigate the cell death and survival of cultured neurons under the same treatment conditions. Glutamate-induced cell death in CX3CR1^+/+^ neurons but not in CX3CR1^−/−^ neurons, and CX3CR1 Ab infusion protected against the death of CX3CR1^+/+^ neurons (Fig. 6B). On the other hand, CX3CR1^−/−^ neurons had a higher survival rate than CX3CR1^+/+^ neurons when exposed to glutamate. Pretreatment with CX3CR1 Ab significantly increased the survival of CX3CR1^+/+^ neurons (Fig. 6C), which indicated that neurons from CX3CR1^−/−^ mice were more resistant to glutamate-induced morphological changes and cell death than those of WT animals.

**Figure 7:**
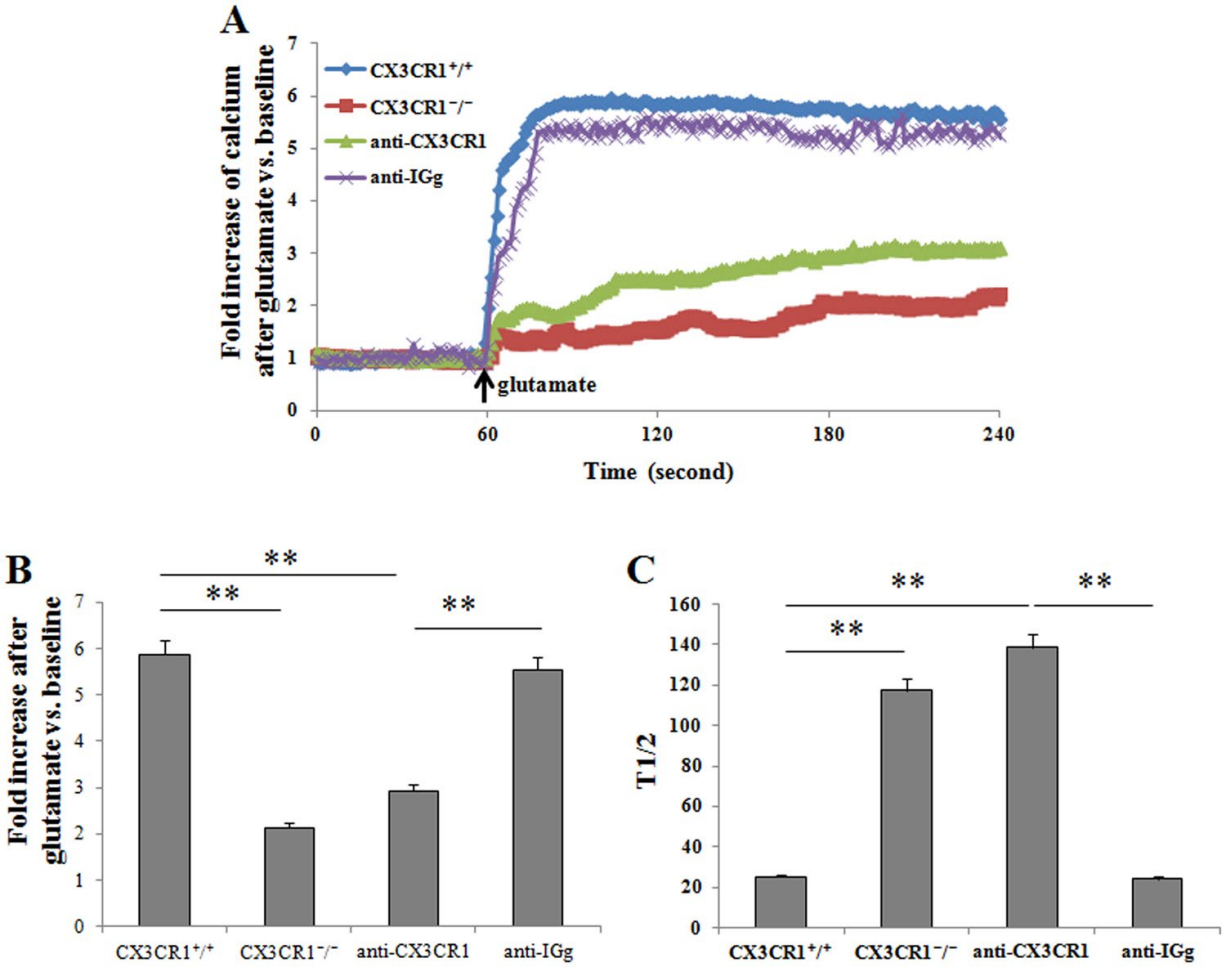
CX3CR1 deficiency reduces calcium influx in glutamate-treated neurons. (**A**) Representative traces of calcium influx were shown. The influx occurred soon after glutamate treatment and reached the peak rapidly, whereas CX3CR1 deficiency and neutralization dramatically decreased the influx. (**B**) The volume of calcium influx was calculated by the fold increase in calcium. Calcium was increased by approximately six-fold in CX3CR1^+/+^ neurons, but only by approximately two-fold in the CX3CR1^−/−^ group. Neutralizing the CX3CR1 could attenuate the calcium increase. (**C**) The time to reach half of the peak calcium concentration was recorded, which represented the rate of calcium influx. CX3CR1 deficiency and neutralization decreased the rate of calcium influx significantly (**p < 0.01). n = 20–30/group.

The early efflux of glutamate occurring immediately after the onset of ischemia is mediated by a calcium-dependent process through activation of voltage-dependent calcium channels. Based on the calcium imaging, calcium was overloaded in glutamate-exposed CX3CR1^+/+^ neurons but not in CX3CR1^−/−^ neurons or anti-CX3CR1 pretreated neurons. Calcium influx in the neurons occurred soon after glutamate treatment and rapidly reached peak calcium concentrations, whereas CX3CR1 deficiency and neutralization of the CX3CR1 dramatically decreased the influx (Fig. 7A). Quantification analysis showed a six-fold increase of calcium in the CX3CR1^+/+^ group, while only an approximately two-fold increase was seen in the CX3CR1^−/−^ group. Neutralization of CX3CR1 can attenuate the calcium increase in CX3CR1^+/+^ neurons (Fig. 7B). The rate of calcium influx was assessed by the time to reach half of the peak calcium concentration. CX3CR1 deficiency and neutralization significantly decreased the rate of calcium infiltration (Fig. 7C). These data indicate that glutamate-induced CX3CR1-dependent ischemic neuron apoptosis is mediated by increased calcium influx.

## Discussion

In this paper, we describe for the first time that neuronal CXCR1 mediates neuronal apoptotic cell death in ischemia. Our principal findings are: (1) CX3CR1 is unregulated in ischemic neurons *in vitro* and in a mouse model of permanent focal ischemia; (2) neuronal CX3CR1 elevation is associated with apoptotic neuronal death; (3) CX3CR1 deficiency protects mice from ischemic brain damage *in vivo*; (4) CX3CR1 deficiency reduces glutamate-mediated excitotoxicity in hippocampal neurons cultured *in vitro* with a mechanism dependent on intracellular calcium. Taken together, our data suggest that CX3CR1 promotes acute brain ischemic pathology by exacerbating excitotoxicity and neuronal apoptosis.

In ischemic stroke, the concept of a penumbra has been proven by studies in patients in whom thrombolytic treatment had reversed ischemia and had decreased the infarct volume^17,18^. Consequently, the tissue in the penumbra is the most clinically relevant target for interventional treatments in acute stroke and the damage may be reversed if treatment is implemented at an early stage. Evidence suggests that excitotoxicity and apoptosis comprise a major route of ischemic neuronal death in the penumbra, while tissue death in the core zone is mainly necrotic^2^. A deep understanding of the mechanisms that promote or prevent recovery is crucial in identifying the means of preventing neuronal death and designing therapies for stroke. Previously, we had found that CX3CR1 deficiency is associated with an improved outcome following transient ischemic brain injury^7^. Our results are consistent with others’ findings in acute brain and spinal cord injury models in that the absence of CX3CR1 significantly reduces ischemic damage and inflammation^8,19,20^. Based on the previous observation that CX3CR1 receptors are exclusively found on microglia and astrocytes^6,10^, we and others had focused on the microglia-neuron communication via CX3CL1/CX3CR1 signaling. In particular, we demonstrated that CX3CR1-mediated microglia activation was associated with neuronal apoptosis in the peri-infarct area at 72 hours after transient middle cerebral artery occlusion (tMCAO) in mice^7^. However, recent findings of the expression of CX3CR1 by hippocampal neurons indicate that these receptors may also be a target for fractalkine produced by microglia and astroglia or adjacent neurons^11^. In this study, we found that CX3CR1 was expressed at low levels in the intact brain and was transiently upregulated in ischemic neurons post-pMCAO. Furthermore, we demonstrated that the upregulated CX3CR1 levels were associated with ischemic neuronal apoptosis in hippocampus at 24 hours after permanent middle cerebral artery occlusion. Our results shed more light on the neurotoxic role of CX3CR1 in ischemia and support the hypothesis that fractalkine and its receptor are involved in a complex network of both paracrine and autocrine interactions between neurons and glia^11,21^.

Our results suggest that neuronal CX3CR1 deficiency may play a neuroprotective role in ischemia, similar to previous reports that CX3CR1 knockout protects against amyloid-β induced neurotoxicity^16^. The neurodegenerative role of CX3CR1 was aslo observed in a rat model of epilepsy^12^, although neuronal CX3CR1 receptors were reported to mediate the neurotrophic effects of fractalkine by activating Akt, a major component of prosurvival signaling pathways, and nuclear translocation of NF-κB, a downstream effector of Akt. This prosurvival signaling pathway protects hippocampal neurons from the neurotoxicity induced by the HIV-1 envelope protein gp120IIIB^11^. Discrepancies in various reports on the neuroprotective versus neurodegenerative functions of neuronal CX3CR1 might be attributed to the regional specificity of its expression in different neurological disorders. In our experiments, we found that both striatal and hippocampal neurons expressed CX3CR1 in accordance with previous reports of selective CX3CR1 expression^16,22^. Since both neurons and microglia appear to express CXCR1, in some contexts particular to the CX3CR1^+/+^ animal model, the result will reflect the complexity of the CX3CL1/CX3CR1 system involving both microglia-neuron and neuron-neuron communication^12,16^.

Excitotoxicity is a primary mechanism of neuronal injury following stroke. It is a form of cell death induced by prolonged neuronal stimulation with high glutamate concentrations, a consequence of impairment of the local energy supply. Glutamate activates glutamate-ionotropic receptors at synaptic and extra-synaptic sites, causing prolonged neuronal depolarization and triggering deregulation of cellular ion homeostasis, mainly intracellular calcium and sodium^23^. In a previous study, we have shown ischemic neuronal excitability and synaptic excitatory transmission using somatic whole-cell current-clamp recording^24^. In this report, we have expanded upon previous researches by showing that CX3CR1 deficiency prevents glutamate-induced excitotoxicity by blocking calcium influx. Neutralizing the CX3CR1 with anti-CX3CR1 antibody can also attenuate glutamate-induced damage. It has been shown that the protective effect of CX3CR1 on gp120-mediated neurotoxicity is through prosurvival signaling of Akt and NF-κB^11^ and that CX3CL1 reduces glutamate-mediated neuronal apoptosis with a mechanism that involves the activation of extracellular-signal-regulated kinases (ERK) and Akt pathways^25^. Additionally, the protective properties of CX3CR1 may be related to the reduction of glutamate-activated currents or CX3CR1-mediated activation of cytosolic pathways. Considerable crosstalk may exist between the proapoptotic and prosurvival signaling pathways mediated by neuronal CX3CR1 and is worthy of further study in the field of ischemic neuronal damage.

**Figure 8:**
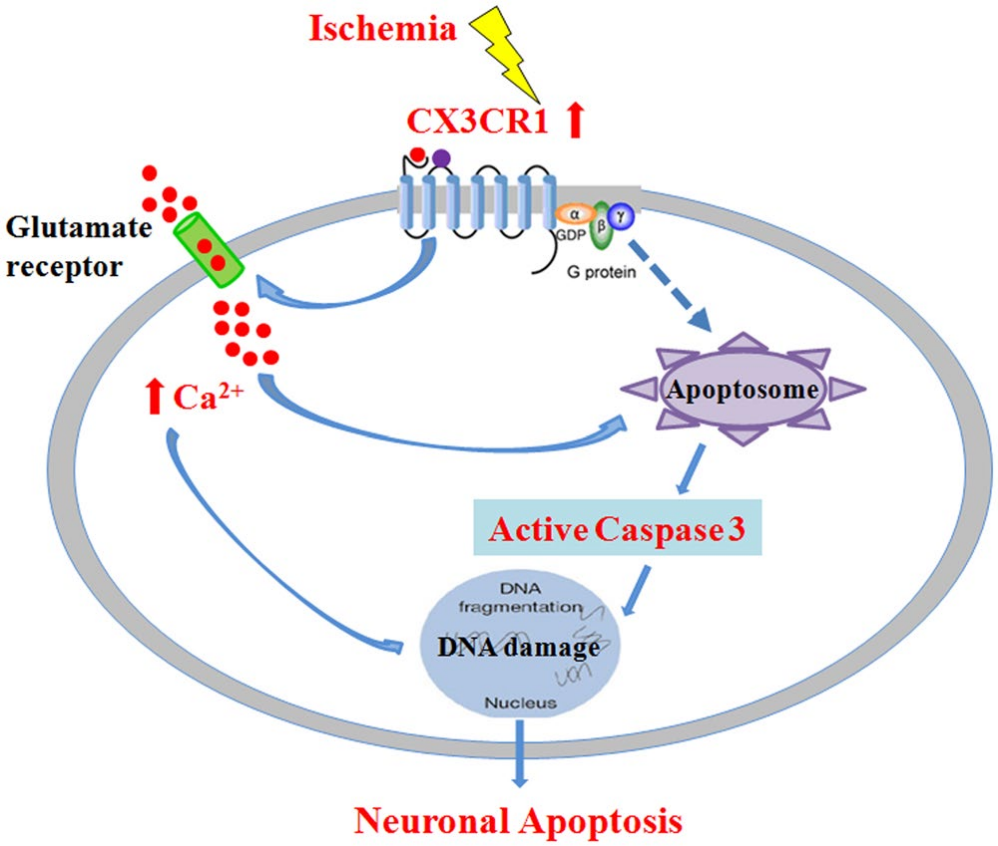
Diagram of the neurodegenerative mechanism of neuronal CX3CR1 in ischemic stroke in mice. Neuronal CX3CR1 mediates the neuronal apoptosis in ischemic stroke through its activation of caspases and glutamate-induced calcium influx. Consequently, CX3CR1 deficiency improved the outcome of mice in a model of ischemic stroke.

In conclusion, we provide valuable insight into the specific mechanisms of ischemic brain injury by suggesting a role for neuron-expressing CX3CR1 in the evolution of ischemic neurons (Fig. 8). Targeting CX3CR1 in order to inhibit apoptosis limits cell death after ischemia, offering a unique approach for stroke therapy.

## Materials and Methods

### Experimental animals

Wild-type (WT) C57BL/6 mice and breeding pairs of CX3CR1^−/−^ mice were obtained from The Jackson Laboratory (Bar Harbor, ME, USA). Knockouts were generated by replacing the second exon of the CX3CR1 gene with the enhanced green fluorescent protein (GFP) reporter gene, and the resulting mice were backcrossed for more than 10 generations to C57BL/6 mice^26^. Cells under the control of the endogenous CX3CR1 locus (microglia, macrophages, dendritic cells, etc.) in homozygous CX3CR1^−/−^ mice were labeled with GFP and lacked CX3CR1 receptor function. All animals were housed in pathogen-free conditions at the animal facilities of the Barrow Neurological Institute, Phoenix, AZ, USA. All experimental procedures were approved by the Institutional Animal Care and Use Committee and performed in accordance with the National Institutes of Health Guide for the Care and Use of Laboratory Animals (NIH publication No. 80–23, revised 1996).

### Permanent middle cerebral artery occlusion

Adult male mice (aged 10 to 14 weeks, weighing 24 to 27 g) were exposed to permanent middle cerebral artery occlusion (pMCAO) with an intraluminal filament^27^. Mice were anesthetized by intraperitoneal injection of ketamine (80 mg/kg) and xylazine (10 mg/kg). In anesthetized mice, the right side of the common carotid artery was exposed and isolated. The middle cerebral artery (MCA) was occluded by inserting a 6–0 surgical monofilament nylon suture with a heat-rounded tip into the internal carotid artery, which was advanced further until it closed the origin of the MCA. During the experiments, MCA blood flow was monitored using a single fiber optic probe (MoorVMS-LDF, Moor Instruments Ltd, UK) adhered onto the skull surface of the core area supplied by the MCA (5 mm lateral and 2 mm posterior from the bregma) before and after clamping the right MCA. Mice that showed a decrease in MCA blood flow of less than 70–80% of the initial value were excluded. Rectal temperature was maintained at 37 ± 0.5 °C by means of a heating blanket and heating lamp throughout the surgery. Sham-operated control mice received the same surgical procedure without inserting a filament.

### CBF, ADC, and T2-weighted images by rodent magnetic resonance imaging (MRI)

MRI experiments were carried out on a 7 T small animal system, 30-cm horizontal-bore magnet, and BioSpec Avance III spectrometer (Bruker Daltonics Inc., CA, USA) with a 116-mm high power gradient set (600 mT/m) and a 72-mm whole-body mouse transmit/surface receiving coil configuration. Anesthesia was induced with 3% isoflurane and maintained with 1.5% isoflurane carried by 1.5 L/min medical air. During MRI scanning, the animal’s respiration was continually monitored by a small animal monitoring and gating system (SA Instruments, Stoney Brook, NY, USA) via a pillow sensor positioned under the abdomen. Mice were placed on a heated circulating water blanket (Bruker, Billerica, MA, USA) to maintain normal body temperature (36–37 °C). Segments of echo-planar imaging (EPI) sequences were used to acquire the apparent diffusion coefficient (ADC) reduction 30 min after pMCAO to assess the damage volume with the following parameters: TR = 5000 ms, TE = 30 ms, 0.5 mm slice thickness without slice gap, field of view (FOV) 2.56 cm × 2.56 cm, matrix: 128 × 128, 18 slices, total scan time of 2 min 20 seconds, and 6 directions of B-field were applied, with b = 100, 200, 400, 600, 800, and 1000 s/mm^2^ along each direction. To assess whether the ischemia was induced successfully, single slice cerebral blood flow (CBF) images were acquired 1 hour after pMCAO using a Continuous Arterial Spin Labeling (CASL) sequence with parameters: TR = 3000 ms, TE = 6.95 ms, segments = 4, slice thickness = 1.5 mm, field of view (FOV) 2.0 cm × 2.0 cm, matrix: 64 × 64, total scan time of 20 min. T2-weighted images were acquired 24 hours after pMCAO to assess the infarction volume using a Rapid Acquisition with Refocused Echoes (RARE) sequence with parameters: TR = 3000 ms, effective TE = 60 ms, RARE factor = 8, 20 slices were acquired with thickness = 0.5 mm, field of view (FOV) 2.56 cm × 2.56 cm, matrix: 128 × 128, total scan time of 3 min 12 seconds. The MRI data were analyzed using the MED × 3.4.3 software package (Medical Numerics Inc., Germantown, MA, USA) on a LINUX workstation.

### 2,3,5-Triphenyltetrazolium chloride (TTC) staining

To confirm the infarction volume determined by the T2-weighted MRI images, TTC staining^28^ was performed immediately after MRI scanning. The animals were quickly anesthetized with isoflurane, and the brains were removed rapidly and frozen at −20 °C for 5 min. Coronal slices were made at 1–2 mm from the frontal tips, and sections were immersed in 2% TTC (Sigma-Aldrich, MO) at 37 °C for 20 min. The presence of infarction was determined by the area that was stained negative with TTC.

### Neurological deficit assessment

Neurological deficit assessments were performed by investigators who were blinded to the experimental groups, as described previously^29^. The following rating scale was used: 0 = no deficit, 1 = failure to extend left forepaw, 2 = decreased grip strength of left forepaw, 3 = circling to left by pulling the tail, and 4 = spontaneous circling.

### Histological analysis and detection of apoptosis

The mice were anesthetized with isoflurane 24 hours after pMCAO. Terminally anesthetized mice were perfused intracardially with saline followed by 4% paraformaldehyde. The fixed brains were embedded in paraffin and cut into 6 µm thick serial coronal slides. Immunohistochemistry was performed with antibodies against mouse CX3CR1 (sc-30030, Santa Cruz, Dallas, TX, USA). Immunolabeling was detected by applying the peroxidase-antiperoxidase procedure with 3, 3′-diaminobenzidine (DAB) as a co-substrate. Prior to immunofluorescence staining, CX3CR1^−/−^ sections were incubated with 10% methanol in PBS to quench endogenous GFP. For double immunofluorescent staining, antibodies against NeuN (MAB377, Millipore, Temecula, CA, USA) and cleaved Caspase-3 (9662 S, Cell Signaling, Danvers, MA, USA) were used to identify apoptotic neurons. For triple immunofluorescent staining, NeuN (MAB377, Millipore, Temecula, CA, USA), CX3CR1 (sc-30030, Santa Cruz, Dallas, TX, USA), and cleaved Caspase-3 (sc-1225, Santa Cruz, Dallas, TX, USA) were used as primary antibodies. Anti-CX3CR1 (sc-30030) specificity has been confirmed by histochemistry staining of brain tissues from the CX3CR1 mutant mice (Supplementary Fig. S2). Respective negative controls that omit primary antibodies and positive controls were applied for each case. The positively stained cells were counted at 20× magnification in matched sections. The results are presented as positively stained cells per mm^2^ within areas measured using ImageJ.1.34vi software (National Institutes of Health).

### Western blot analysis

Ipsilateral and contralateral hemispheres of pMCAO mice were homogenized in 150 mM NaCl containing 50 mM Tris-HCl (pH 7.2), 1% Triton X-100, and complete mini protease inhibitor mixture tablets (Roche Diagnostics GmbH, Mannheim, Germany). After centrifugation, the supernatants were used for immunoblot analysis. Anti-CX3CR1 (diluted 1: 300; ab8021, Abcam, Cambridge, MA, USA) and anti-beta Actin (diluted 1: 1000; sc-47778, Santa Cruz, Dallas, TX, USA) were used as the primary antibodies. The specificity of anti-CX3CR1 (ab8021) was verified by using CX3CR1 deficient mice (Supplementary Fig. S2). Immunodetection was performed and quantified using the Li-Cor Odyssey CLx Imaging System (LI-COR Inc., Lincoln, NE, USA) according to the protocol provided by the manufacturer.

### Primary hippocampal neuronal cultures

Primary hippocampal cultures were prepared from the brains of WT and CX3CR1^−/−^ mouse pups P1. After careful dissection of the diencephalic structures, the meninges were removed, and the hippocampi were chopped and digested with 0.025% trypsin in Hank’s balanced salt solution (HBSS) at 37 °C for 20 min. Cells were mechanically dissociated and plated at a density of 2.5 × 10^5^ in poly-L-lysine coated plastic 24-well dishes in serum-free Neurobasal medium supplemented with B27, 0.5 mM L-glutamine and 100 μg/ml gentamicin. Two days after plating, the cells were treated with 2.5 μΜ cytosine arabinoside, an inhibitor of DNA replication (Sigma-Aldrich, St. Louis, MO, USA) for 3 days to prevent mitotic, non-neuronal cell growth^30^. Under these conditions, an average neuronal purity of 92% can be achieved. Neuronal cultures were kept at 37 °C in 5% CO2 for 10–11 days *in vitro* with a biweekly medium replacement by removing half of the old media and replacing it with the same volume of fresh media^31^.

### Glutamate excitotoxicity

In primary CX3CR1^+/+^ (WT) and CX3CR1^−/−^ hippocampal cultures, the conditioned medium was removed and stored for later use; neurons were washed and treated with glutamate (50 μM glutamate + 10 μM glycine, 30 min) in modified Locke’s buffer (without MgCl_2_ plus 1 μM glycine) to stimulate all types of glutamate receptors. When necessary, cells were pre-treated with polyclonal CX3CR1 Ab (10 μg/ml, 30 min; sc-30030, Santa Cruz, Dallas, TX, USA) or rabbit IgG (10 μg/ml, 30 min; BA-1000, Vector laboratories, Burlingame, CA, USA) in culture medium; drugs were present during and after the glutamate challenge. After treatment, cells were re-incubated in the original conditioned medium for 18–20 hours.

Glutamate excitotoxicity towards neurons was measured using the lactate dehydrogenase (LDH)-based Cyto Tox 96 non-radioactive cytotoxicity assay kit (TOX7, Sigma-Aldrich, St. Louis, MO, USA) in accordance with the manufacturer’s protocol. Glutamate-treated cultured neurons were pelleted by centrifugation at 250 × g for 4 min. The supernatant was transferred to a new 96-well plate, and the reconstituted substrate mix was added. The absorbance was measured at 490 nm. Each assay was tested in triplicate. The percentage of specific lysis was determined as follows: (experimental release-target spontaneous release)/ (target maximum release-target spontaneous release) × 100. The maximum release was determined by detecting the absorbance of the target cells lysed with LDH assay lysis solution.

The viability of glutamate-treated cultured neurons was tested by an MTT assay. Specifically, 5 mg/ml MTT was added 1:10 to the cultures and incubated for 2 hours; the medium was aspired, then the cells were treated with DMSO and incubated at 37 °C for 10 min. Samples were then analyzed with a microplate reader at 490 and 630 nm to subtract the background. The results were expressed as a percentage (%) of viable cells in the control cultures.

### Calcium imaging

After being cultured for 10 days, primary neuronal cells on coverslips were washed in warmed HBSS. They were then incubated in 3 μM Fluo-3-acetoxymethyl ester (Fluo-3/AM) in HBSS at 37 °C for 20 min. Coverslips were washed again with HBSS and placed on the stage of a laser scanning confocal microscope (LSCM, Carl Zeiss, Oberkochen, Germany).

For live cell calcium imaging, cells were excited with 488 nm light from an argon laser, and an emission of 530 nm was detected. The cells were scanned once every second for 240 seconds. After 60 seconds of baseline recording in HBSS, cells were exposed to glutamate (50 μM glutamate + 10 μM glycine) in HBSS for 180 seconds^32^.

The intensity of the fluorescence in each cell, which represented the concentration of intracellular calcium, was recorded. The baseline fluorescence was normalized to 1, and the data were presented as the fold increase over the baseline.

### Statistical analysis

The results are presented as the means ± SEM. Statistical differences between two groups were evaluated by the two-tailed unpaired Student’s *t*-test. Multiple comparisons were performed with two-way ANOVA accompanied by Bonferroni’s post hoc test. Values of p < 0.05 were considered significant.

### Data availability

All data generated or analyzed during this study are included in this article.

## Electronic supplementary material

Supplementary Information
