# Supplementary material for: Ischemia-induced Neuronal Cell Death Is Mediated by Chemokine Receptor CX3CR1
Source: Sci Rep. 2018 Jan 11;8:556. doi: 10.1038/s41598-017-18774-0 (PMC5765008; doi:10.1038/s41598-017-18774-0)
Supplement: Supplementary file 1 — Supplementary Information [file 41598_2017_18774_MOESM1_ESM.pdf]

Ischemia-induced Neuronal Cell Death Is Mediated by Chemokine Receptor CX3CR1

Running title: CX3CR1 deficiency prevents ischemic neuronal death

Jinkun Wang[1,*], Yan Gan[1,2,*], Pengcheng Han[2], Junxiang Yin[2], Qingwei Liu[3], Soha Ghanian[2,4], Feng Gao[1], Guanghui Gong[1] & Zhiwei Tang[1,2]

[1]Department of Neurosurgery, The First Affiliated Hospital of Kunming Medical University, Kunming, Yunnan, China

[2]Department of Neurology, Barrow Neurological Institute, St. Joseph's Hospital and Medical Center, Phoenix, AZ, USA

[3]Department of Radiology, Barrow Neurological Institute, St. Joseph's Hospital and Medical Center, Phoenix, AZ, USA

[4]Brown University Warren Alpert Medical School, Providence, RI, USA

*These authors contributed equally to this work.

Correspondence and requests for materials should be addressed to Z.T. (email: tangzhiwei7755@hotmail.com)

Supplementary Figures

Figure S1

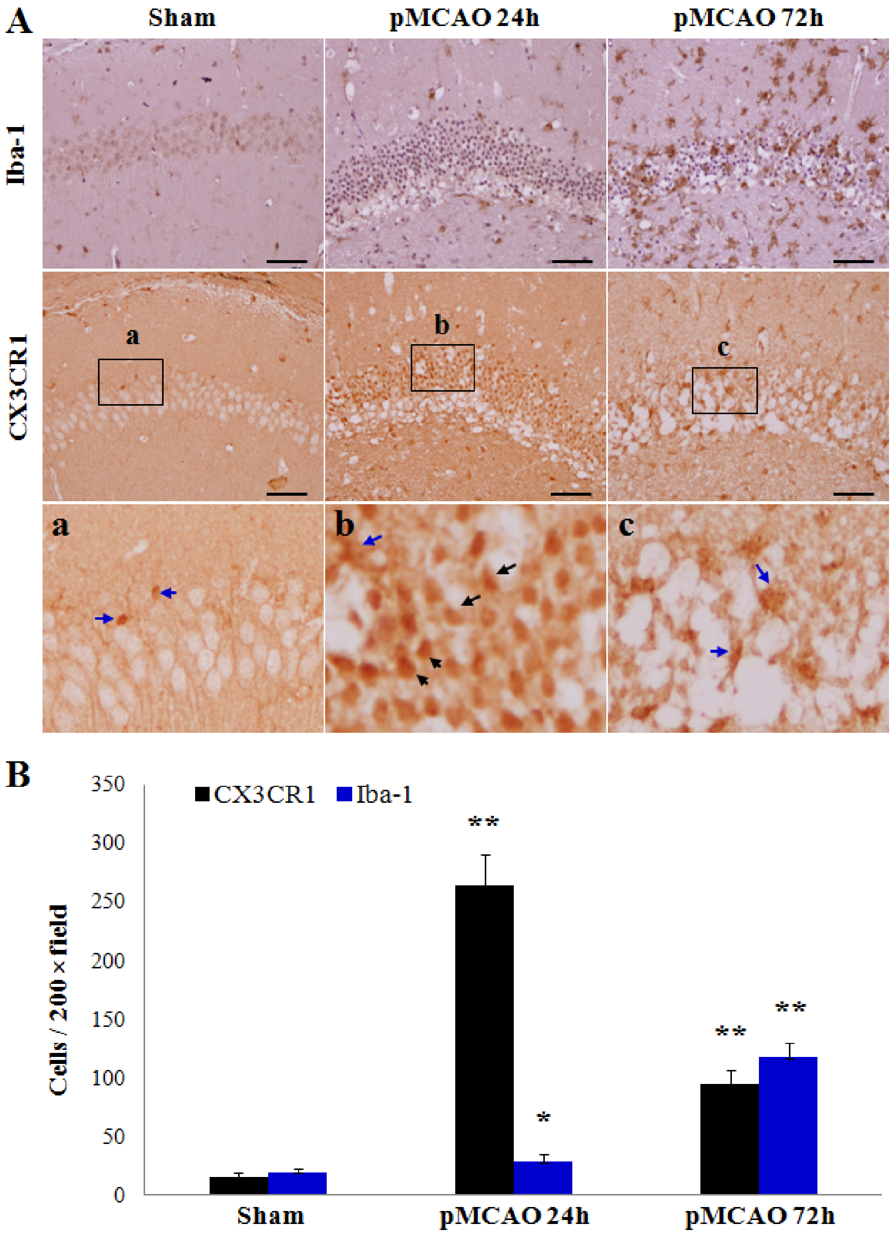

Fig. S1. Expression of CX3CR1 on microglia in the infarct brain of pMCAO mice. (A) Iba-1 and CX3CR1 levels were determined in the hippocampus of the ipsilateral hemisphere of C57BL/6 mice 24 and 72 hours after pMCAO by histochemistry staining (upper and middle panel). Sham-operated animals served as controls. Images at the bottom show magnified views

of the boxed areas in the middle panel. The black and blue arrows indicate neuron-like cells and microglia-like cells, respectively. The CX3CR1 positive cells at 72 hours after occlusion are mainly microglia-like cells. Scale bars = 50 am, n = 4/group. **(B)** Semi-quantification analysis showed that the number of CX3CR1 positive cells significantly increased 24 hours and decreased 72 hours after pMCAO (**$p < 0.01$). Iba-1 positive cells were seen to slightly increase at 24 hours (*$p < 0.05$) and peaked at 72 hours (**$p < 0.01$) post-pMACO. 72 hours after occlusion the number of CX3CR1 positive cells was close to that of Iba-1 positive cells, demonstrating confirmatory evidence to the morphology findings. Cells were counted in 5 randomly chosen 200× magnification fields on five sections in four replicate mice per group in three separate experiments.

Figure S2

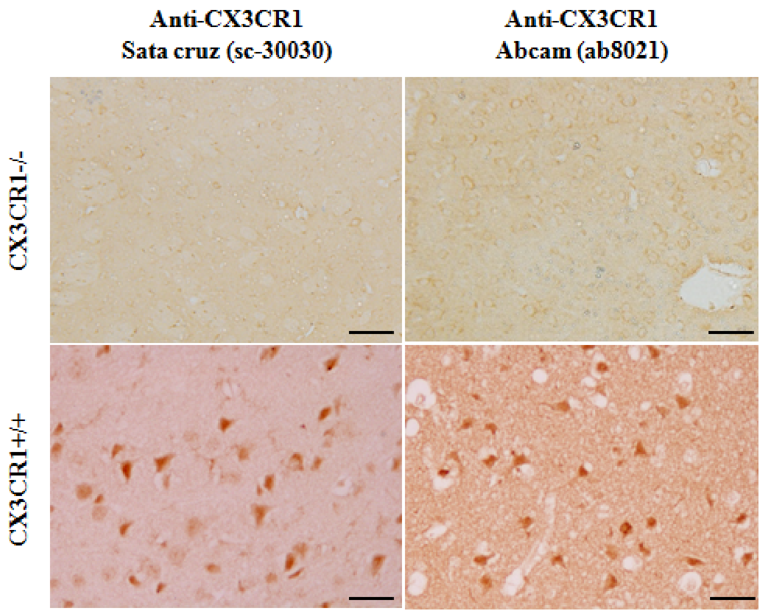

Fig. S2. Specificity of the CX3CR1 antibodies was verified by histochemistry staining of brain tissues from the CX3CR1 deficient mice. Anti-CX3CR1 (sc-30030) and anti-CX3CR1 (ab8021) were used for immunostaining and western blot in the current study. Both did not show immunoreactivity to brains tissues of CX3CR1$^{-/-}$ mice (upper panel), but positively reacted with CX3CR1$^{+/+}$ tissues from wild type mice (bottom panel). Scale bars = 50 μm, n = 4/group.
